# Shifting North American drug markets and challenges for the system of care

**DOI:** 10.1186/s13033-021-00512-9

**Published:** 2021-12-20

**Authors:** R. Michael Krausz, Jean N. Westenberg, Nickie Mathew, George Budd, James S. H. Wong, Vivian W. L. Tsang, Marc Vogel, Conor King, Vijay Seethapathy, Kerry Jang, Fiona Choi

**Affiliations:** 1 Department of Psychiatry, Faculty of Medicine, University of British Columbia, Vancouver, BC Canada; 2 Complex Pain and Addiction Service, Vancouver General Hospital, Vancouver, BC Canada; 3 BC Mental Health & Substance Use Services, Provincial Health Services Authority, Vancouver, BC Canada; 4 University of Basel Psychiatric Clinics, Basel, Switzerland; 5 Division of Substance Use Disorders, Psychiatric Services of Thurgovia, Münsterlingen, Switzerland; 6 Victoria Police Department, Victoria, BC Canada; 7 Addictions and Concurrent Disorders Research Group, Institute of Mental Health, UBC, David Strangway Building, 5950 University Boulevard, Vancouver, BC V6T 1Z3 Canada

**Keywords:** Drug markets, Drug policy, Fentanyl, Overdose, Public health crisis

## Abstract

Drug markets are dynamic systems which change based on demand, competition, legislation and revenue. Shifts that are not met with immediate and appropriate responses from the healthcare system can lead to public health crises with tragic levels of morbidity and mortality, as experienced Europe in the early 1990s and as is the case in North America currently. The major feature of the current drug market shift in North America is towards highly potent synthetic opioids such as fentanyl and fentanyl analogues. An additional spike in stimulant use further complicates this issue. Without understanding the ever-changing dynamics of drug markets and consequent patterns of drug use, the healthcare system will continue to be ineffective in its response, and morbidity and mortality will continue to increase. Economic perspectives are largely neglected in research and clinical contexts, but better treatment alternatives need to consider the large-scale macroeconomic conditions of drug markets as well as the behavioural economics of individual substance use. It is important for policy makers, health authorities, first responders and medical providers to be aware of the clinical implications of drug market changes in order to best serve people who use drugs. Only with significant clinical research, a comprehensive reorganization of the system of care across all sectors, and an evidence-driven governance, will we be successful in addressing the challenges brought on by the recent shifts in drug markets.

## Introduction

Due to the ongoing overdose crisis in Canada and the United States (US), key components in the system of care must be reconsidered, including governance and treatment paradigms. Akin to the HIV pandemic, appalling mortality rates are getting far too little attention and the healthcare system is not responding as needed. Remarkable is the amount of attention and resources going towards the COVID-19 pandemic in comparison to the overdose crisis, despite the fact, that e.g., in British Columbia (BC), the epicenter of the Canadian overdose crisis, the numbers of people dying by overdose was more than 50% higher in relation to COVID-19 in 2020 [1, 2]. Treatment capacity for people who use drugs (PWUD), which was insufficient to begin with, has further decreased to make room for COVID-19 relief. For example, in response to physical distancing protocols, Insite, one of Vancouver’s Supervised Consumption Sites (SCS), reduced its capacity from 24 to 6. BC wide, the number of people accessing SCSs dropped by almost 50%, from 853,619 overdose prevention site visits in 2019 to 570,619 in 2020 [3]. Moreover, known triggers for substance use and relapse such as stress and isolation have all increased as a result of the pandemic. This has contributed to increased prevalence or severity of substance use disorders and harms related to substance use [4, 5]. To worsen an already dire situation, business closures, border closures, and physical distancing directives have led to changes in the drug markets, accelerating the increased presence of higher potency synthetic opioids such as fentanyl and fentanyl analogues [6].

Health conditions such as high-risk substance use are mainly interpreted as individualized behavioral consequences based on psychological, genetic or biological underpinnings. However, the dynamics of drug markets significantly influence the economic and social factors that frame individual substance use behaviors. It is important to understand the dynamic changes in demand, competition, legislation and revenue of drug markets in order to plan for appropriate health care adaptations or reforms.

## Overdose crisis overview

**Fig. 1:**
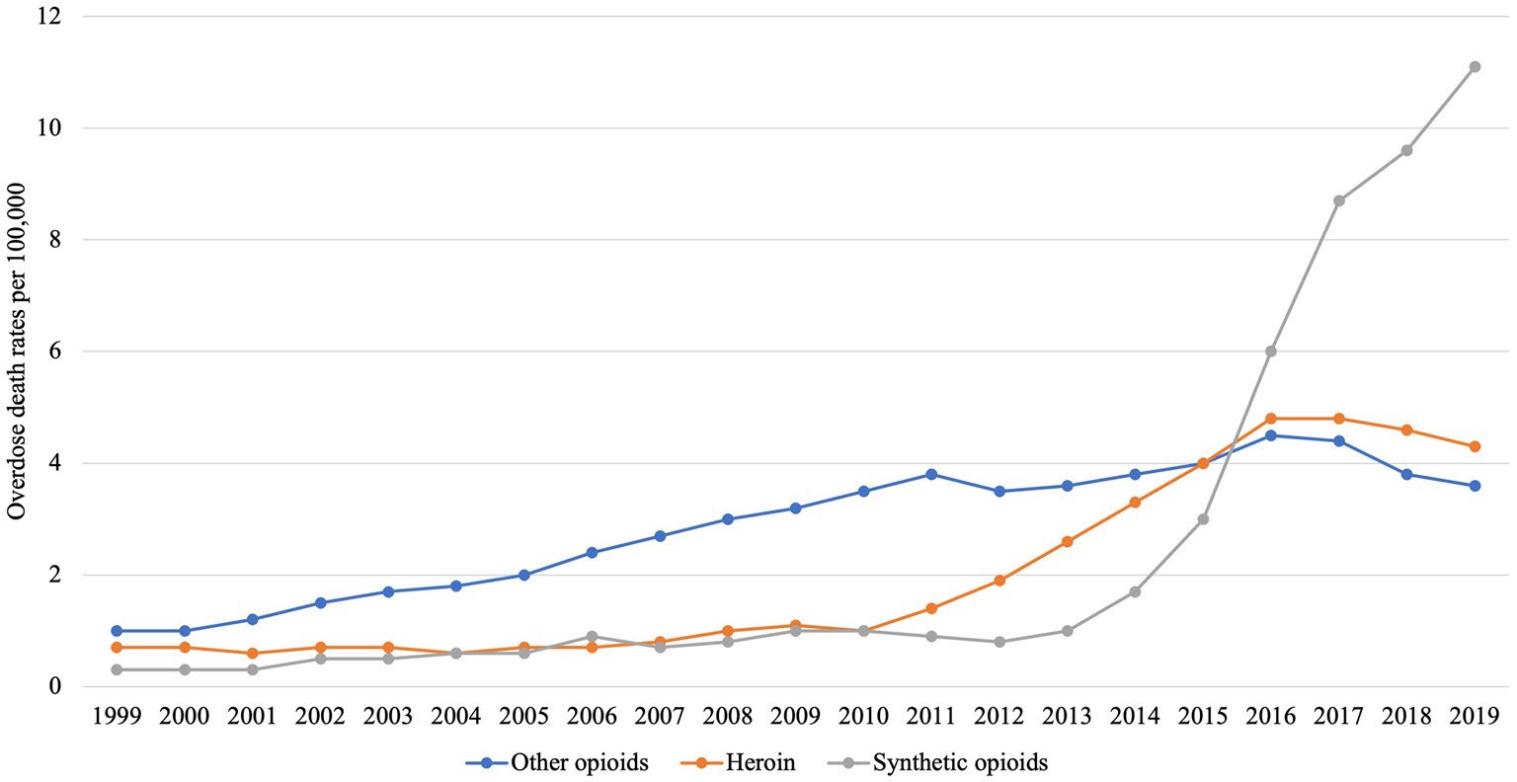
The three waves of the overdose crisis: opioid overdose deaths by type of opioid. Drug poisoning deaths classified using ICD-10 underlying cause of death codes (X40–X44, X60–X64, X85 and Y10–Y14) and by drugs involved as immediate or contributory causes of death: heroin (T40.1), synthetic opioids, most importantly fentanyl (T40.4) and opioids other than methadone, opium, heroin and synthetic opioids (T40.2). Data accessed from the Centers for Disease Control and Prevention, National Center for Health Statistics. Multiple Cause of Death 1999–2019 on CDC WONDER Online Database, released in 2020. Data are from the Multiple Cause of Death Files, 1999–2019, as compiled from data provided by the 57 vital statistics jurisdictions through the Vital Statistics Cooperative Program. Accessed at http://wonder.cdc.gov/mcd-icd10.html on Nov 21, 2021 2:55:14 PM

Between 1999 and 2019, the overdose crisis in North America has been described in three distinct waves (Fig. 1) [7]. The first wave was attributed to the significant increase in the medical prescription of opioids for post-operative use and pain management, which placed the American and Canadian opioid consumption per capita among the highest in the world [8]. This was spurred on by pressures and marketing tactics from large pharmaceutical companies towards physicians in prescribing opioids such as OxyContin [9, 10], but also by insufficient “opioid stewardship” spanning legal systems and healthcare systems, most notably pain treatment and substance use treatment. The second wave began in 2010 with a rapid increase in street heroin-related overdoses, while the third wave, which began in 2013, marks the appearance of synthetic opioids and the rise of synthetic opioid-related deaths [7]. These synthetic opioids, such as fentanyl and its analogues, K2/Spice (cannabinoid), and those with cryptic acronyms like 25i-NBOMe (hallucinogen), were all originally conceived in legitimate laboratories for proper scientific and therapeutic purposes, but their formulas were then exploited and manufactured. Moreover, to stay ahead of the law, their molecular structures are being consistently altered which make the drugs' effects unpredictable [11, 12]. A potential fourth wave may be gathering force due to increases in stimulant use (especially methamphetamine), entwined with high availability and use of highly potent opioids [13].

## Phases of change related to synthetic opioids

Since the increased spread of synthetic opioids in the third wave of the overdose crisis, the landscape of drug markets in the United States and Canada has gone through several phases, which have continued to exacerbate the public health crisis and overdose related fatalities (Table 1). What began as a traditional drug market with occasional fentanyl use (Phase 0) grew to widespread contamination with imported fentanyl (Phase 1), advancing towards local production of potent opioids and omnipresence of fentanyl in the drug market (Phase 2).

**Table 1:** The phases of development in the North American drug market related to fentanyl use

Phase	Timeline	Significant features	Overdose	Outcomes
0—traditional drug market: occasional fentanyl use	Before 2013	Repurposing of fentanyl patches; fentanyl use in small groups	Occasional fentanyl-related overdose deaths	Local problems
1—growing market: contamination of street drugs	2013–2018	Import of fentanyl from Asia to North America and Mexico	Significant increase in overdose deaths; synthetic opioids surpass heroin and prescription opioids	Regional problems, especially West Coast of Canada
2—developed market: locally produced fentanyl	2019 to present	Interruption of overseas fentanyl import; precursor import and local fentanyl production; additional COVID-19 impacts	Historically high numbers of opioid overdose deaths; almost exclusive fentanyl-related	High availability of fentanyl; new routes of application; intentional fentanyl use

### Phase 0—“traditional” drug market (before 2013)

Introduction of fentanyl into the drug market started as a small phenomenon related to the diversion of fentanyl patches, before organized importation of highly potent synthetic opioids began in North America and Mexico [14, 15]. This continues to be the present reality of most European countries which are not experiencing the same widespread fentanyl use and increase of drug overdoses compared to North America [16–18].

There were also a few isolated large-scale illicit fentanyl laboratories in operation. For example, one located in Toluca, Mexico had been in operation for several years and has been linked to a surge in overdose deaths between 2005 and 2007 in the Mid-West of the United States [19]. This specific laboratory was eventually shut down by Mexican Authorities in 2007, and was surprisingly not immediately replaced by another. Illicit fentanyl production was mostly halted across North America, with China emerging as a source country.

### Phase 1—contamination of street drugs in a mixed market (2013–2018)

Several factors lead to fentanyl's increasing prevalence in the drug market, which was being almost exclusively imported from China [20]. The most important advantage of fentanyl is its potency, approximately 70 times more potent in depressing respiratory rate that of heroin or morphine [21]. Less fentanyl is needed to attain “euphorigenic” effects, which greatly benefitted traffickers due to the lowered risk of interception by authorities. Another advantage of fentanyl was its synthetic nature, allowing for eventual streamlining of laboratory production and lower price of overall production. Fentanyl was therefore systematically mixed or “cut” with heroin, and dealers were able to sell greater amounts of so-called “heroin” for higher profits [20]. This eventually became the standard business model. At the time, most users were not aware of the presence of fentanyl, which resulted in consecutive overdose experiences among PWUD [22, 23]. In Canada, various Organized Crime Groups (OCG) were and are responsible for fentanyl importation from China and distribution within the country, starting in British Columbia and moving towards the East. In the US, Mexican cartels have played a similar role.

The contamination of the drug supply was reflected in substance use patterns and drug seeking behaviors. Changes in demand were reported from traditional heroin seekers to individuals who used mixed opioids (heroin and fentanyl) and individuals who used fentanyl primarily [24]. The routes of administrations also diversified from patches and tablets to injection, utilizing at-home “cooking” techniques to convert oral tablets into liquids for intravenous use. Inhaling also became an important route of administration, especially in the BC market, creating an illusion of safety for users because of the pharmacokinetic parameters of inhalation, including lower peak plasma concentrations relative to injection [25]. This method of administration makes it more attractive to new users especially, who prefer to not inject for various reasons. Nevertheless, the BC Coroner Service report that among all overdoses fatalities, deaths related to inhaling climbed from 28.3% in 2016 to 40.8% in 2017, and remain above 40% to this day [26].

Nevertheless, fentanyl gradually took over the drug market. The Drug Enforcement Agency (DEA) and the Canadian Border Service Agency both reported substantial increases in seizures of illicitly manufactured fentanyl [27, 28]. For instance, in the United States, the total number of fentanyl reports submitted to forensic laboratories increased by 65% from 2016 to 2017 [28]. As the level of fentanyl in the drug markets gradually increased from 2013 to 2018, so did opioid-related overdose fatalities [29].

### Phase 2—omnipresence of fentanyl in the drug market

Fentanyl-containing products, which was mostly localized and distributed in patches across North America up until 2019, then became omnipresent. Contaminated street drugs that were laced with fentanyl gave way to new market trends, including PWUD desiring pure fentanyl. Given fentanyl’s increasing influence in the drug market, policy responses attempted to address the prolific international fentanyl trafficking networks.

### Phase 2a—legal action in China against fentanyl production and exportation (2019)

In May 2019, China prohibited the production, sale and exportation of all fentanyl class-drugs except for some authorized firms which the Chinese government granted special licenses [30]. The influx of fentanyl from China to North America noticeably declined. Canadian Customs and Border Protection reported that seizures of fentanyl directly shipped from China dropped from over 116 kg in 2017 to less than 200 g in 2019 [30].

### Phase 2b—import of precursors from Asia to Mexico and British Columbia (2019 to present)

The interruption of the fentanyl supply led to an adaption in production and supply strategies. As a result of China’s class scheduling of fentanyl, precursors in the production of fentanyl-like opioids, which did not fall under restrictions, began being traded. Precursors like N-Phenethyl-4-piperidinone (NPP) and 4-anilino-N-phenethylpiperidine (4-ANPP) are both used as intermediates in the synthesis of fentanyl. After production of these precursors in Asia and exportation to North America and Mexico, they were then used for fentanyl local production. International regulations then banned the two primary precursors, NPP and 4-ANPP, but alternate precursors began being produced and shipped [30].

### Phase 2c—trading through Mexico (2019 to present)

Using imported precursors from China, Mexican Trans-National Criminal Organizations (TCO), primarily the Sinaloa Cartel and the Jalisco New Generation Cartel, are able to produce immense volumes of high-grade fentanyl. The production and subsequent distribution of their fentanyl utilizes established laboratories and trading routes already used for other substances. The fentanyl is bundled in large quantities and smuggled across the Southwest Border, often with shipments that also contain bundles of Mexican heroin and Columbian cocaine. The bundles are distributed across the US by regional drug trafficking groups and street gangs who will then adulterate the fentanyl with cutting agents to increase the volume, or mix it with other illicit drugs, before it reaches end users [28].

### Phase 2d—trading through British Columbia (2019 to present)

As the importation and availability of fentanyl decreased because of the ban introduced by the Chinese Government, Canadian OCGs, primarily in British Columbia, turned to the domestic production of fentanyl. Though laboratories had been heavily involved in the production of methamphetamine, operations have been repurposed and capacity has been augmented for local fentanyl production, utilizing drug synthesis knowledge, laboratory equipment and clandestine locations previously employed in methamphetamine production [31].

Domestic production has resulted in a much greater availability of fentanyl on the drug market, as the volume of fentanyl produced per laboratory in BC is known to be much more than the volume imported from overseas. For instance, the Royal Canadian Mountain Police (RCMP) located a large illicit drug-manufacturing site in Surrey, BC in early May 2021, containing 37 kg of chemicals associated with fentanyl production, with a potential estimated finished yield of 26 kg of pure fentanyl per week [32]. In the year prior, between 2020 and 2021, the Canadian Border Services Agency report having seized roughly 7.4 kg of fentanyl [33]. As a result of this increased availability, OCG and those within the distribution network, from street gangs to individual actors, do not need to adulterate the fentanyl to the same degree as was necessary previously, when the only source was China and the volume available was much lower. This results in much greater purity (close to 85%) when compared to the pre-domestic production period, though the potency has remained similar. PWUD are therefore consuming much purer fentanyl, and this has been reflected in post-mortem toxicology screens. In the past year, approximately 13% of overdose deaths had extreme fentanyl concentrations (exceeding 50 µg/litre), as compared to 8% a year prior [1]. Moreover, carfentanil, an elephant tranquilizer about 10,000 times more potent than morphine and 30–100 times more potent than fentanyl, has been detected in 113 overdoses in the first 6 months of 2021, already far exceeding the 2020 total of 65 [1].

## Clinical consequences

The changes of the drug market in US and Canada have led to a significant shift in patterns of use and a dramatic increase in drug overdose cases and fatalities, especially in BC [1]. The situation is not currently identical in all regions of North America, with provinces such as British Columbia and Alberta as well as states such as California and Florida experiencing the highest number of overdose deaths in Canada and the United States respectively [35]. Nevertheless, the changing landscape of synthetic opioid use has led to changes both in clinical observations and considered interventions when treating patients for overdoses or harm reduction through opioid agonist treatment (OAT). It is important for policy makers, health authorities, first responders and medical providers to be aware of the clinical implications of the changes in the drug market in order to best serve PWUD.

### 1. Increasing significance of fentanyl

Fentanyl and its derivates have gained an immense importance in North America. Before 2012, there was only sporadic use of fentanyl in different parts of the world, including Europe. Cooking fentanyl patches and injecting the fluid was rare, and led to multiple fatalities in Munich, Germany [36]. Injection or inhalation of fentanyl was not widespread, and the use of contaminated street drugs was unintentional in the beginning. In North America, this pattern of use has now changed towards intentional use of fentanyl and fentanyl-seeking behaviors. Fentanyl has become dominant based mainly on four factors: cheaper price, increased availability, high potency, and very intense onset with a short intoxication period, relative to other opioids [37]. For instance, fentanyl's price and availability has made it more attractive to inhale, which has increased its use among to diverse groups of PWUD, not only PWID.

### 2. Fentanyl as drug of choice

Drug checking to identify fentanyl in street drugs and avoid its consumption is something of the past. Today, a growing number of PWUD utilize drug checking services to find fentanyl, which has become the drug of choice for some [38, 39]. A main reason for the shift from unintentional to intentional fentanyl use may be an attempt to regain control. Many PWUD have been exposed to unintentional fentanyl use over the last couple of years, which has resulted in an increase in tolerance, thereby making it difficult for PWUD to continue using less-potent opioids such as heroin or oral medications such as oxycontin [24]. The high tolerance in current PWUD means that individuals are forced to seek higher potency opioids, now only found in fentanyl and fentanyl derivatives. For example, a pattern of use largely seen between 2016 and 2019 was the mixing of intravenous heroin with fentanyl, also called ‘fentanyl with legs’ as the heroin kicks in while the rush of fentanyl fades away. However, this practice has waned as heroin is increasingly difficult to find at the street level. There is also an increase in mixing fentanyl with sedatives, including benzodiazepines like alprazolam and diazepam, and chemically related drugs like etizolam, in order to similarly provide “legs” to the fentanyl. Responding to mixed fentanyl-benzodiazepine overdoses is a complex, challenging medical intervention dealing with two classes of drugs simultaneously [40].

Moreover, though patterns of use are driven by neurobiological mechanisms and market forces, they are also based on individual decision-making, personal responsibility, and choice, limited as they may be in such circumstances. For instance, from an individualistic perspective, one's risk of overdose may be in fact more manageable if one seeks fentanyl, since seeking pure heroin within a market contaminated by high potent opioids may make it almost impossible to appropriately manage one’s risk of overdose. These are aspects that are to a large degree ignored in research and clinical contexts, despite being important considerations for the development of effective interventions. To provide better alternatives, an effective strategy needs to also include microeconomic and behavioural economic analyses of individual substance use, which there is a paucity of research on [41, 42].

### 3. Inappropriate and insufficient medical care

Adverse events related to fentanyl use (e.g., seizures and overdoses) happen on a regular basis and are leading to skyrocketing numbers of emergency calls (over 27,000 in BC in 2020) [43]. In acute care settings, patients with intravenous fentanyl use face the problem of quick and intense withdrawal because the treatment protocols are not adapted to their needs [44–47]. Many of these patients’ opioid tolerance is too high for routine OAT options such as methadone, morphine, and breakthrough medications such as hydromorphone [48, 49]. Current hospitals mainly use transdermal formulations of fentanyl and inpatient populations are not routinely prescribed intravenous OAT such as heroin-assisted therapy, which is currently regulated in 8 European countries and Canada and has been proven more clinically effective and cost-effective than oral methadone for this target group [50, 51]. Consequently, many patients facing unbearable withdrawal leave against medical advice despite untreated physical illness [52, 53].

### 4. Stimulants in the developed market

Stimulants (i.e., methamphetamine) continues to be among the most prevalent substances in the American and Canadian illicit drug market [31]. Methamphetamine use has increased significantly among people with an existing opioid use disorder, and there have been significant increases in drug combinations of fentanyl with methamphetamine and cocaine [54, 55]. It is highly possible that as stimulants and depressants are often used in tandem, the arrival of potent opioids like fentanyl has necessitated increasingly potent stimulants through importation and distribution [31]. Recent findings have suggested a potential fourth wave of high mortality may be gathering force in North America involving methamphetamine and cocaine, which may be a new determinant in the ongoing public health crisis [13]. Though stimulant use has been prevalent, treatment options have never truly been available and needs related to stimulant use never been appropriately addressed [56]. There is a need to recognize the explosion in methamphetamine and cocaine related mortalities, and to develop necessary treatment options for stimulant withdrawal and crisis management which are further complicated by fentanyl and high potent opioids [1].

## Discussion

Drug markets are similar to other internationally organized markets. They follow the rules of supply and demand to optimize revenue. The introduction of highly potent opioids has followed these economic principles, with fentanyl and its derivatives outperforming heroin and prescription opioids in North America. For now, the main substance of choice is fentanyl, but this may change again depending on how it will address the risk/revenue ratio.

Addressing a public health crisis requires effective evidence-based healthcare governance and an amalgamation of necessary structures and resources. The experiences with the COVID-19 pandemic provide great learning opportunities for what to avoid and what works, like the benefit of using innovative technologies, investments in new interventions (e.g., COVID-19 vaccine) and the organization of a coherent clinical trajectory.

Finding solutions to the current wave of the overdose crisis requires significant clinical research. For instance, we know little about the user perspective. This would be essential knowledge to enable successful engagement in care and appropriate early intervention. Another topic is the effective response of the healthcare system. How can a treatment be effectively provided and how can appropriate psychosocial and pharmacological approaches be improved and adapted to new challenges? Would free access to psychotropic substances, including high potent opioids, decrease mortality and reduce harm? [58, 59]. Is the unsupervised distribution of high potent psychotropic substances, such as hydromorphone or prescription stimulants, an additional risk? The concept of fentanyl-assisted treatment, analogous to heroin-assisted treatment, has been gaining traction among clinical and academic forums, and large scale clinical research efforts exploring such pragmatic approaches are an immediate priority [60].

In the coming months and possibly years, three main scenarios are to be considered. The first scenario (worst-case) would be the dominance of high potent opioids with a system of care unable to respond. This would inevitably lead to further increases in overdose related mortality. A second scenario (better) would be a slowing down of overdose mortality rates, based on some effective health care measures such as rapid access to quality OAT treatment with high potent opioids like heroin-assisted treatment, universal access to supervised consumption sites, and universal access to psychosocial treatments for anybody at high-risk of overdose or suicide [61, 62]. Such an integrated clinical trajectory would need substantial changes from the current course of action. The third scenario (best) would be having the healthcare system and decision makers addressing the shift in the drug markets with a response comparable to that used during the HIV pandemic. This would include a critical reconsideration of all components involved in the current approach to substance use care, including the legal framework (e.g., decriminalization of use), a structure for continuity of care, and building non-existent components like prevention, early intervention, youth specific systems.

## Conclusions

We are experiencing a historic shift in the North American drug markets towards highly potent synthetic opioids. Fentanyl and fentanyl analogues are available in different forms, mixed into the street supply or consumed directly as the preferred drug of choice, and are leading to a dramatic increase in mortality across the continent. Research efforts must direct more focus towards the macroeconomic conditions of the drug market and the microeconomics of individual substance use, which are critical to the planning and development of appropriate treatments, services and resources. The only way to stop this is with significant efforts to create comprehensive systems of prevention and treatment, using innovative measures of intervention and immediate accessibility.

## Data Availability

N/A.

## Abbreviations

HIV: Human immunodeficiency virus
COVID-19: Coronavirus disease of 2019
PWUD: People who use drugs
SCS: Supervised consumption sites
OCG: Organized crime groups
DEA: Drug enforcement agency
OAT: Opioid agonist treatment
US: United States
BC: British Columbia
